# A target-protection mechanism of antibiotic resistance at atomic resolution: insights into FusB-type fusidic acid resistance

**DOI:** 10.1038/srep19524

**Published:** 2016-01-19

**Authors:** Jennifer H. Tomlinson, Gary S. Thompson, Arnout P. Kalverda, Anastasia Zhuravleva, Alex J. O’Neill

**Affiliations:** 1 School of Molecular and Cellular Biology, Garstang Building, University of Leeds, Leeds, UK, LS2 9JT; 2 Astbury Centre for Structural Molecular Biology, Faculty of Biological Sciences, University of Leeds, Leeds, UK, LS2 9JT

## Abstract

Antibiotic resistance in clinically important bacteria can be mediated by proteins that physically associate with the drug target and act to protect it from the inhibitory effects of an antibiotic. We present here the first detailed structural characterization of such a target protection mechanism mediated through a protein-protein interaction, revealing the architecture of the complex formed between the FusB fusidic acid resistance protein and the drug target (EF-G) it acts to protect. Binding of FusB to EF-G induces conformational and dynamic changes in the latter, shedding light on the molecular mechanism of fusidic acid resistance.

Antibiotic resistance is an evolving crisis that threatens to undermine our ability to treat bacterial infection^1^. To effectively tackle this issue, a comprehensive understanding of bacterial resistance to antibiotics will be crucial. In particular, it will be important to achieve a detailed knowledge of the molecular mechanisms involved, not least because such information could potentially inform strategies to inhibit these mechanisms and thereby rejuvenate the clinical efficacy of antibacterial drugs whose activity has become compromised by resistance.

Whilst there has been extensive study into the mechanisms of antibiotic resistance, and a broad understanding now exists, some notable gaps in our knowledge remain. One of these concerns the phenomenon of target protection by protein-protein interaction, wherein an antibiotic resistance protein directly binds the drug target protein and acts to protect it from the inhibitory effects of an antibiotic. To date, only two examples of such a resistance mechanism have been identified to mediate clinically significant antibiotic resistance; FusB-type resistance to fusidic acid (FA), and Qnr-mediated resistance to fluoroquinolones^2^,^3^. In neither case is there an understanding at the molecular level of the structural basis for the protein-protein interaction or of the mechanism by which the interaction gives rise to resistance. Here we describe a series of studies to gain insight into both of these aspects for the target protection mechanism of FA resistance mediated by FusB-type proteins.

FA is widely used as a topical agent to treat staphylococcal skin infection, and is also one of the few remaining oral antibiotics effective against methicillin-resistant *Staphylococcus aureus* (MRSA)^4^,^5^. FA acts by interfering with correct functioning of elongation factor G (EF-G)^6^,^7^, the protein responsible for catalyzing translocation of peptidyl-tRNA from the A site to the P site of the ribosome during protein synthesis^8^. Once translocation has occurred, EF-G ordinarily dissociates from the ribosome, vacating the A site for the next incoming aminoacyl-tRNA species. In the presence of FA, the drug binds to EF-G on the ribosome and inhibits its release, thereby preventing further protein synthesis and leading to growth arrest^6^,^7^.

Recent years have seen a dramatic increase in resistance to FA in clinical strains of *S. aureus* and other staphylococci^9^. In most FA-resistant strains, resistance is the result of horizontal acquisition of determinants that encode FusB-type proteins^9^,^10^,^11^,^12^,^13^. Members of the FusB family, the best studied of which is FusB itself, are small (~25 kDa) proteins that bind EF-G with a 1:1 stoichiometry^14^ and protect it from the inhibitory effect of FA. X-ray crystallographic studies have determined the 3D structures of two representatives of the FusB family^14^,^15^, revealing a two-domain protein with an unusual zinc-binding fold in the C-terminal domain. Through direct interaction with EF-G, FusB promotes disassembly of the FA-stalled post-translocation complex, thereby rescuing protein synthesis^14^,^16^. FusB has also been shown to increase turnover of EF-G on the ribosome in the absence of FA, implying that it acts to accelerate the conformational rearrangement within EF-G necessary for dissociation from the ribosome^14^. The ability of FusB to drive EF-G release explains why it mediates resistance to FA; this effect will directly counter that of FA, which is acting to prevent dissociation of EF-G from the ribosome. How FusB might achieve this effect remains an outstanding question.

To enable an understanding of the mechanism of FusB-mediated target protection, an appreciation of the molecular details of the interaction occurring between the resistance protein and the drug target will be required. Although individual structures of *S. aureus* EF-G^17^,^18^,^19^,^20^,^21^,^22^ and FusB-type proteins^14^,^15^ have been determined (Fig. 1), the structure of the two proteins in complex has not, and attempts to produce crystals of the FusB•EF-G complex for structural determination by X-ray diffraction have to date not proven successful (*unpublished data*). As a consequence, only limited information exists regarding the architecture of the complex. NMR minimal chemical shift perturbations (CSPs) and mutagenesis mapping have established that regions within the C-terminal domain of FusB represent the primary site of interaction with EF-G^14^,^23^. The corresponding site of interaction on EF-G is less well defined, although FusB has been shown to bind with comparable affinity to an EF-G fragment (EF-G_C3_) lacking the N-terminal domains I and II, implying that the binding site resides within domains III-V^14^.

Here, we employed solution NMR to elaborate a structural model of the FusB•EF-G complex. In addition to delineating the architecture of the resistance protein in complex with the drug target, this approach revealed conformational and dynamic changes occurring in EF-G upon complexation that provide an explanation for the action of FusB on EF-G, and in turn, the mechanism of FusB-mediated resistance to FA.

## Results

### Establishing a model system for NMR characterization of the FusB•EF-G complex

The ability to effectively study protein complexes by NMR is strongly influenced by molecular size, with smaller systems achieving greater accuracy and enabling the use of a wider range of methodological approaches. In view of this, and of the equivalent FusB-binding properties of EF-G (77 kDa) and the truncated version of the protein, EF-G_C3_ (35 kDa)^14^, we chose the latter for studying the FusB•EF-G complex by NMR. To ensure that truncation of EF-G did not perturb the structure of the protein, we compared ^15^N-TROSY-HSQC spectra of the truncated and full-length proteins in both apo and FusB-bound states. Spectra from EF-G and EF-G_C3_ overlaid well (Supplementary Fig. 1), revealing no significant chemical shift differences apart from isolated effects along the interfaces with domains I and II (domains missing from the truncated version). Amide residual dipolar coupling (RDC) measurements, which provide a good measure of the local structure for each residue and allow the determination of the relative orientation of domains and secondary structure elements, established that EF-G_C3_ fits well to the crystal structure of *S. aureus* EF-G (PDB ID 2XEX)^17^ (Supplementary Fig. 2) (Q factor of 0.39), indicating that no interdomain changes occur upon truncation of the protein. RDCs measured in FusB also confirmed a good fit between the crystal structure^15^ and solution structure of this protein (Supplementary Fig. 2) in the apo state (Q factor of 0.41). Thus, the solution structures of apo FusB and apo EF-G_C3_ do not differ substantially from the published crystal structures, and EF-G_C3_ represents a suitable surrogate for the full-length protein in interaction studies with FusB.

### Towards determination of a large, multidomain complex structure using NMR

Structural determination of large protein complexes using NMR spectroscopy remains a considerable challenge. This reflects the fact that the number and types of structural restraints that can be determined are limited, and consequently models must be based on sparse data sets. Although a variety of approaches have been utilized to obtain such structural data using different types of sparse data sets^24^,^25^,^26^,^27^, most rely on expensive and time-consuming selective labelling of methyl groups, and no uniformly successful approach has yet been detailed^28^. Here, we combined several well-established techniques^24^,^26^,^29^,^30^,^31^,^32^,^33^ to drive docking calculations in a streamlined protocol that did not require selective methyl labelling or nuclear Overhauser effect (NOE) data. This three step approach, detailed below, allowed us to produce a structural model of the 60 kDa FusB•EF-G_C3_ complex and identify structural changes occurring in the binding partners at sub-domain level upon complex formation.

#### Step 1: Defining the structure of the individual protein domains using RDC and solvent PRE (paramagnetic relaxation enhancement) measurements

To assess structural changes within individual domains upon complexation, amide RDCs measured in the complex within expected secondary structure elements were fitted to the crystal structures of each domain independently. Amide RDCs measured in FusB bound to EF-G_C3_ produced a good fit for both the N- and C-terminal domains of the FusB crystal structure^15^ (Supplementary Fig. 3), showing that neither domain undergoes significant internal structural changes upon binding to EF-G_C3_. RDC measurements on domain V of EF-G_C3_ bound to FusB also produced a good fit to the EF-G crystal structure^17^, indicating no substantial conformational changes occur within this domain upon complexation. RDCs for domain IV fit well to most of the domain, but poor fits for the first α-helix and part of the second suggest that the orientations of these helices change upon binding of FusB (Supplementary Fig. 4). Structural changes within domain III could not be assessed due to resonance broadening upon binding to FusB (*see below*). To model the structural changes within domain IV upon complexation, the crystal structure of the domain, excluding these α-helices, was fixed and the helices refined in orientation against the RDCs as rigid bodies hinged within flexible loops using Xplor-NIH^34^. Solvent PREs, in which a paramagnetic species is introduced into the bulk solvent to measure the degree of solvent exposure of residues within a protein^26^, were measured in EF-G_C3_ bound to FusB and utilized as restraints in these calculations^29^. The inclusion of these restraints assisted in determining structural rearrangements and maintenance of a compact structure, resulting in a refined structural model of domain IV with a good fit to all secondary structure RDCs (Supplementary Fig. 4). To assess the quality of the fit to the RDCs, a Q_free_ was calculated using eight repeats and a random selection of 10% values in the free set. Refinement of the helices in domain IV reduced the Q_free_ from 0.53 to 0.42, confirming that the refinement improves the fit to the RDCs.

#### Step 2: Determining domain orientations in the complex using RDCs and solvent PREs

To determine whether the relative domain orientations in each protein were altered upon complexation, the previous independent fits of amide RDCs within secondary structure elements to each domain were compared to the fit for the whole of FusB and EF-G_C3_. The fit to the RDCs for both FusB and EF-G_C3_ was poorer than that achieved taking each domain separately, with higher Q factors than the independent fits (Supplementary Figs 3 and 4); furthermore, these separate fits produced different orientations for the alignment tensors of each domain, indicating a change in relative domain orientation on complexation. To characterize this domain reorientation, the individual domains (treated as rigid bodies hinged at the domain boundaries) were refined in orientation against the RDCs during restrained molecular dynamics simulated annealing calculations using Xplor-NIH^34^. In the case of EF-G_C3_, these calculations included restraints produced by solvent PREs. As a result of broadening of signals within domain III of EF-G_C3_ upon complexation, no restraints could be determined for this domain; this domain was therefore left unrestrained during structure calculations to avoid biasing the structural model. Structure calculations suggested a modest reorientation of the N-terminal domain of FusB relative to the C-terminal domain, which gave a better fit to the RDCs (Q factor of 0.40, compared with 0.59 for the apo crystal structure). The Q_free_, calculated as above, reduced from 0.61 to 0.55 upon refinement, confirming the improvement in the fit to the RDCs. The refined, FusB-bound structure of EF-G_C3_ produced by these calculations exhibited a better fit to the RDCs than the apo crystal structure (Supplementary Fig. 4) (Q factor of 0.21, compared with 0.43 for the apo crystal structure). The Q_free_, calculated as above, reduced from 0.48 to 0.39 during refinement, validating the fit to the reoriented conformation. This refinement step therefore produced models of the domain orientations in both proteins in the complex that agreed well with experimental restraints.

#### Step 3: Docking of the individual refined protein structures to produce a structural model of the complex

To complete the structural model of the FusB•EF-G_C3_ complex, we considered together chemical shift perturbation (CSP) data identifying the primary binding sites, RDC data to restrain the relative orientations of the two proteins and PRE data within EF-G_C3_ bound to FusB MTSL tagged at residues 19, 26 and 150 (as detailed in materials and methods and supplementary information) for residues showing an I_ox_/I_red_ (the ratio of peak intensity in the MTSL tagged and diamagnetic samples) greater than 0.9 or less than 0.1 (yielding long range distance restraints). These data were used to drive semi-rigid docking of the two proteins in the conformations determined above using HADDOCK^35^. Docking calculations produced a unique solution with an average RMSD of 0.68 Å over 200 structures that was consistent with all of our data. The use of only PREs with I_ox_/I_red_ >0.9 or <0.1 in docking calculations allowed the remaining, intermediate PREs to be used to verify the model by comparison with calculated values. Theoretical PREs calculated from the final model were therefore compared to experimental values for all residues, including those omitted from the docking calculation, to confirm the structural model was consistent with PRE data (Supplementary Fig. 5).

### A structural model of the FusB•EF-G_C3_ complex

Utilizing the approach outlined above, we produced an unambiguous structural model of the FusB•EF-G_C3_ complex. This model reveals that both FusB and EF-G_C3_ undergo interdomain reorientation upon complexation, with EF-G_C3_ also undergoing intradomain rearrangement within the vicinity of the FusB binding site, as detailed below.

**Figure 2:**
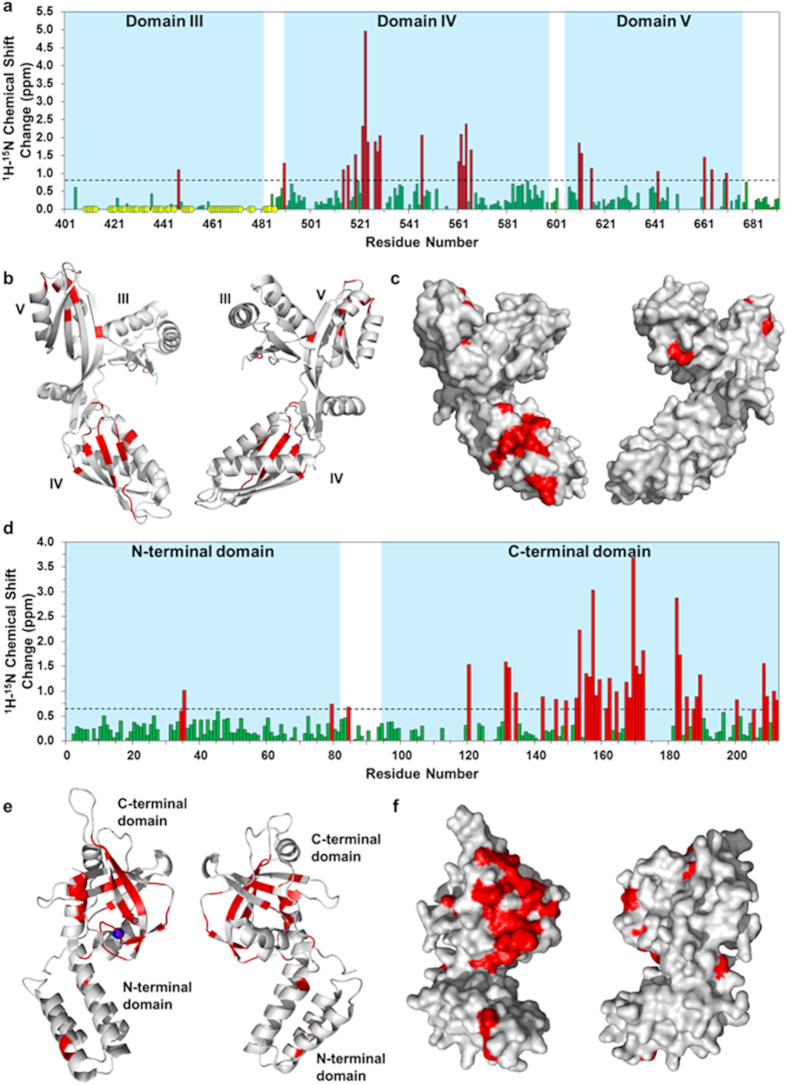
Identification of residues that form the binding interface in EF-G_C3_ and FusB, and mapping of these binding sites onto the corresponding protein structures. (**a**) Combined ^1^H and ^15^N chemical shift changes in EF-G_C3_ upon binding FusB calculated only for those residues assigned in both apo and FusB bound spectra. CSPs considered significant (>1.0 ppm) are shown in red. Residues for which peaks disappear from the spectrum upon complexation are shown as yellow circles. (*N* = 155) (**b**,**c**) Residues showing significant CSPs are indicated in red on 180° rotated views of the crystal structure of apo EF-G_C3_ displayed as a ribbon diagram (**b**) and in surface view (**c**). Significant perturbations predominantly form a patch on one side of domain IV, and represent the primary site of interaction with FusB. (*N* = 167) (**d**) Combined ^1^H and ^15^N chemical shift changes in FusB upon binding to EF-G_C3_ using direct measurement for residues assigned in both spectra and minimal shift changes for all residues assigned only in the apo spectrum assuming the closest bound state peak represents the same residue. CSPs considered significant (>0.6 ppm) are shown in red. (**e,f**) Residues showing significant CSPs are indicated in red on the crystal structure of apo FusB as a ribbon diagram (**e**) and in surface view (**f**). Significant perturbations predominantly form a patch on one side of the C-terminal domain, and represent the primary site of binding to EF-G.

CSP analysis established that the primary FusB binding site forms a patch on one face of domain IV of EF-G_C3_ that centers on β-strand IV_III_ (Fig. 2a–c). The corresponding binding site for EF-G_C3_ on FusB was previously mapped to the C-terminal domain of the latter;^14^ here, this binding site was more precisely delineated (Fig. 2), revealing that the interaction interface involves the second β-sheet and last α-helix of the C-terminal domain of FusB, fully encompassing the zinc coordination site, and extending to the boundary between the N- and C-terminal domains (Fig. 2d–f). Additional, smaller and less well-defined regions of CSPs were observed on the β-sheet of domain V of EF-G_C3_ (Fig. 2a–c) and in the N-terminal domain of FusB, suggesting a secondary site of interaction between EF-G_C3_ and FusB (*see below*).

**Figure 3:**
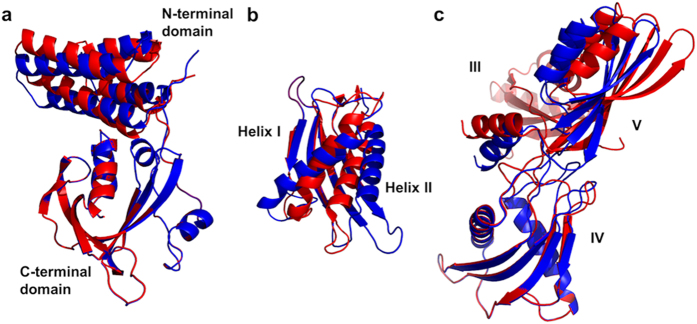
Conformational change occurring in FusB and EF-G_C3_ upon complexation. The crystal structures of the apo states are shown in red, with the states present in the complex shown in blue. (**a**) Conformational change in FusB upon binding to EF-G_C3_ (structures aligned via the C-terminal domains). (**b**) Conformational change in domain IV of EF-G_C3_ upon binding to FusB (structures aligned via the domain IV β-sheet). (**c**) Conformational change in domain IV–V orientation in EF-G_C3_ upon binding to FusB (structures aligned via domain IV). The position of domain III in the FusB bound state of EF-G_C3_ is modelled on the position in the apo state crystal structure, since signal broadening resulted in a lack of data to restrain this domain (*see text*).

Refinement of the FusB and EF-G_C3_ crystal structures to RDC data in the complex revealed that both proteins undergo domain reorientation upon complexation. The N-terminal domain of FusB reorients by 9° relative to the C-terminal domain, although the internal structure of each domain remains unaltered. Domain V of EF-G_C3_ undergoes a 21° reorientation with respect to domain IV upon complexation (Fig. 3). There is also some rearrangement of the internal structure of domain IV of EF-G_C3_ upon complexation; helix IV_I_ becomes tilted upon FusB binding, with the C-terminal end of the helix moving away from the FusB binding site and towards the second helix, whilst helix IV_II_ tilts away at the N-terminal end to compensate for this movement (Fig. 3).

**Figure 4:**
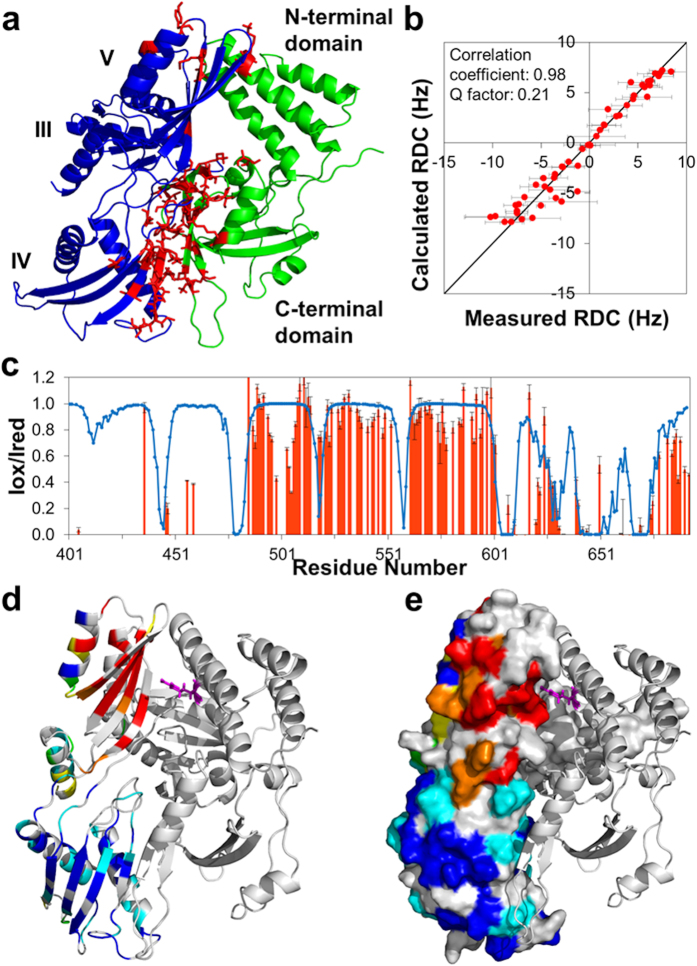
Structural model of the FusB•EF-G_C3_ complex. (**a**) The complex of FusB (green) bound to EF-G_C3_ (blue), determined by docking the crystal structures of the proteins informed by NMR CSPs, RDCs and PREs. Residues showing significant CSPs are shown as red sticks. The position of domain III of EF-G_C3_ is taken from the 2XEX crystal structure as signal broadening meant that no data to restrain the position of this domain could be obtained. (**b**) The correlation between amide RDC measurements in secondary structure regions of EF-G_C3_ bound to FusB and those calculated from the complex structure (*N* = 52). (**c**) Correlation between amide ^1^H PREs measured in EF-G_C3_ bound to FusB-R_19_C-MTSL (red bars) and those calculated from the complex structure (blue line) (*N* = 123). (**d**,**e**) PREs to FusB-R_19_C-MTSL (FusB shown as a cartoon, R_19_ as magenta sticks) in EF-G_C3,_ shown as a cartoon in (**d**) and a surface representation in (**e**). Residues with I_ox_/I_red_ > 0.9 are shown in blue, 0.7–0.89 in cyan, 0.5–0.69 in green, 0.3–0.49 in yellow, 0.1–0.29 in orange and <0.1 in red. Residues for which there was no PRE data are shown in grey.

Docking calculations identified a structure in which EF-G_C3_ and FusB interact via two binding sites; the C-terminal domain of FusB binds the β-sheet of EF-G_C3_ domain IV, and the N-terminal domain of FusB interacts with the β-sheet of EF-G_C3_ domain V (Fig. 4). The domain reorientations within both FusB and EF-G_C3_ result in sufficient movement of the N-terminal domain of FusB and domain V of EF-G_C3_ relative to the primary binding sites to allow the domains to interact while avoiding the steric clashes that occur between the apo structures when docked in this orientation. The structure of the complex places in close proximity the residues in domain V of EF-G_C3_ and the N-terminal domain of FusB that show otherwise unexplained CSPs; since these CSPs were not included in the docking parameters, this provides additional corroborating evidence for the validity of the structure. The structure of FusB•EF-G_C3_ we present here is consistent with all available data (Fig. 4), including that not used to inform calculation of the structure^14^,^15^,^23^, and reveals clearly how FusB and EF-G interact.

### Binding of FusB alters the conformational flexibility of domain III in EF-G

**Figure 5:**
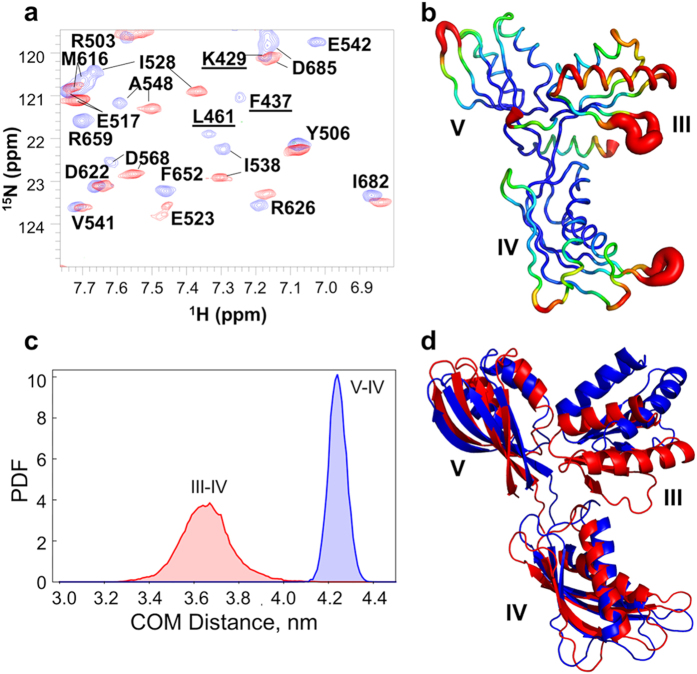
Domain III of EF-G_C3_ undergoes dynamic changes upon binding to FusB. (**a**) Overlay of the ^15^N-TROSY-HSQC of apo EF-G_C3_ (blue) and EF-G_C3_ bound to FusB (red). Peaks from domain III are identified by underlined labels and are absent from the spectrum determined in the presence of FusB. (**b**) Backbone RMSF per residue determined from molecular dynamics simulations of EF-G_C3_. Domain III shows greater dynamic motions than domains IV and V, particularly within the first helix and β-strand. (**c**) Probability density functions for distance distributions between the centres of mass for domains III-IV and IV–V showing greater variation in the distances for domains III–IV, suggesting domain III is flexible. (**d**) The extent of motions of domain III relative to domains IV and V identified by molecular dynamics simulations. Structures are aligned on both domains IV and V, and residues in regions linking the three domains were ommited from the analysis.

A considerable number of the resonances visible in the ^15^N-TROSY-HSQC of apo EF-G_C3_ were lost upon complexation with FusB, without the concomitant emergence of new peaks (Fig. 5). In all, the latter spectrum lacked ~35% of the expected peaks from throughout EF-G_C3_, most of which represent signals from domain III. Whilst 75% of the potentially assignable residues in domain III could be successfully assigned in apo EF-G_C3_, only 14% were identified in the complex spectrum (Supplementary Fig. 6), indicating that most domain III resonances became broadened in the complex. This loss of domain III resonances from the spectrum could not be attributed to the overall broadening of resonances that occurs due to the increase in molecular size; we saw no similar loss of resonances from domains IV and V, both of which gave comparable peak intensities to domain III in the apo state. These data therefore indicate that binding of FusB to EF-G_C3_ prompts domain III to undergo conformational exchange with dynamic motions on a μs-ms timescale. The fact that signals from throughout domain III are lost upon complexation, while those from the remainder of EF-G_C3_ are retained, suggests that the observed effect involves rearrangements throughout the domain rather than movement of the domain as a rigid body. Since domain III borders the site at which EF-G was truncated to yield EF-G_C3_, we sought confirmation that the observed effect was not an artefact of the truncated protein; amide spectra from full-length EF-G confirmed that this was not the case, with domain III signals also becoming lost from EF-G upon complexation with FusB (Supplementary Fig. 1). Although signals from domains I and II were not assigned in spectra of full length EF-G, these domains contributed approximately the expected number of peaks to spectra measured in both the apo and FusB-bound states, suggesting that the FusB-induced dynamics observed are limited to domain III. Furthermore, very few chemical shift changes were observed in resonances from domains I and II, and of those that were, all fell below the level considered significant in EF-G_C3_ upon FusB binding. Therefore, no significant conformational change appears to occur in domains I and II upon FusB binding.

To explore the conformational flexibility associated with domain III of EF-G_C3_ and to investigate whether FusB is likely to enhance the intrinsic dynamic properties of domain III or induce additional dynamics in the complex, we performed molecular dynamics simulations using GROMACS^36^. These simulations suggest that domain III of EF-G is more dynamic than domains IV and V (Fig. 5), with the first helix of the domain showing the greatest per residue RMSF throughout the simulation, a movement consistent with the broadening of resonances observed throughout domain III on FusB binding. Analysis of the distances between centers of mass for each domain throughout the simulations also indicates that domain III moves relative to domains IV and V. Principle component analysis of simulations suggest that domain III is capable of a range of motions, including a flexing of the domain and rotations relative to domains IV and V (Fig. 5). In addition, although domain III is largely ordered in the crystal structure of *S. aureus* EF-G^17^ and RDC data support its ordered conformation in the apo state in solution, several of the crystal structures of *T. thermophilus* EF-G show domain III to be partially disordered with areas of missing electron density^18^,^20^,^37^, further supporting the idea that this domain is capable of dynamic motion. FusB is therefore likely to enhance the intrinsic dynamic properties of domain III.

Collectively, our results establish how FusB interacts with EF-G, and show that this interaction prompts conformational rearrangement in the latter, along with changes in the conformational flexibility of domain III. As discussed below, the ability of FusB to modulate the conformational properties of EF-G can explain the ability of FusB to drive release of EF-G from the ribosome, both in the presence and absence of FA.

## Discussion

With the aid of a new NMR protocol for the structural characterization of large multidomain protein complexes, we have determined the architecture of the ~60 kDa complex formed between an antibiotic resistance protein (FusB) and a truncated form of the drug target it acts to protect (EF-G). This protein-protein interaction involves two regions of contact between the binding partners, with the C- and N-terminal domains of FusB interacting with domains IV and V of EF-G, respectively (Fig. 4). The structure of the FusB•EF-G complex differs substantially from that previously modelled using unrestrained *in silico* docking^14^, in which FusB-type proteins were proposed to make contact with EF-G exclusively through their C-terminal domain, and adopt a binding orientation ~180 degrees from that presented here. Determination of the structural basis for the interaction between these proteins now provides a solid foundation for understanding the mechanism of FusB-type resistance to FA.

In our earlier study, we considered it unlikely that FusB-type proteins bring about FA resistance by interfering with binding of the drug to EF-G^14^. This idea was based on (i) the observation that FusB is able to accelerate release of EF-G from the ribosome both in the presence and absence of FA, making it unnecessary to invoke a direct effect of the protein on drug binding to explain resistance, and (ii) the aforementioned *in silico* docking model of the two proteins, which suggested that FusB-type proteins bind at a site on EF-G removed from the FA binding site. The structural model presented here underscores this idea, confirming that the binding sites of FusB and FA are distinct and non-overlapping, and indeed that they involve different domains of EF-G (whilst FusB binds domains IV and V, FA recognizes a site between domains II and III^21^). In line with this, the closest approach that FusB makes to the FA binding site in the structure of the complex presented here is 17 Å distant.

**Figure 6:**
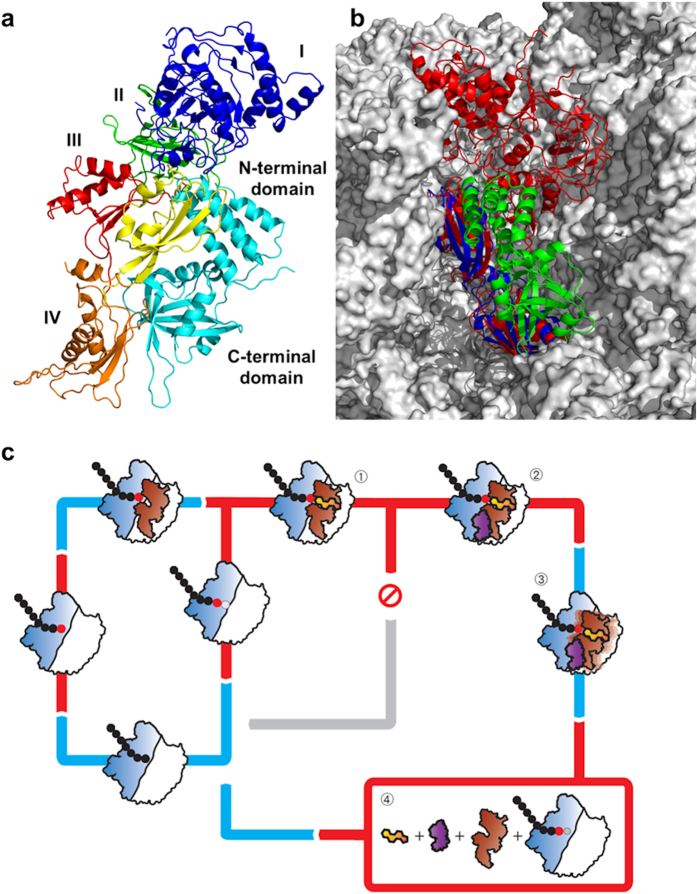
Structure of the FusB•EF-G complex, and the mechanism of FA resistance. (**a**) A model of FusB binding to full length EF-G produced by aligning the FusB•EF-G_C3_ structural model with the crystal structure of ribosome bound EF-G. EF-G is colored by domain and FusB is shown in cyan. (**b**) The model of the FusB•EF-G_C3_ complex docked (by sequence alignment of domains IV and V) onto EF-G bound to the ribosome in the post-translocational state. EF-G_C3_ is shown in blue, FusB in green, EF-G bound to the ribosome in red, and the ribosome in grey. (**c**) Suggested mechanism of FusB-mediated resistance to FA. EF-G (brown) binds to the ribosome (blue and white) after peptide bond formation between the A and P site amino acids and mediates translocation to the P and E sites. EF-G then dissociates making the A site available for binding of the next tRNA. In the presence of the drug, FA (yellow) binds to EF-G on the ribosome in the post-translocation state (1) and stalls protein synthesis by preventing EF-G release. Binding of FusB (purple) to EF-G in stalled complexes (2) induces a conformational change in domains IV and V of EF-G and a change in the dynamics of domain III (3). Either this conformational change is sufficient to promote release of EF-G or the dynamics within domain III allow the C-terminal domains of EF-G to more readily adopt the conformation relative to domains I and II required for release, without the requirement for transmission of conformational change from domains I and II. This results in dissociation of EF-G from the ribosome, thereby allowing protein synthesis to continue (4).

We also speculated previously that FusB-type proteins might drive dissociation of EF-G from the ribosome by directly competing with the latter for EF-G binding, an idea again based on the earlier *in silico* docking studies, which implied that binding of EF-G to the ribosome and FusB represent mutually exclusive events^14^, and on the high affinity of FusB-type proteins for EF-G^14^. Examination of the FusB•EF-G_C3_ complex structure determined in this study, in conjunction with published structures of EF-G bound to the ribosome^21^,^38^, indicates that the FusB binding site on EF-G would likely be fully accessible even when the latter is bound to the ribosome (Fig. 6). Consequently, our present study finds no evidence in support of the idea that FusB might drive release of EF-G by direct competition with the ribosome.

Instead, our findings point to FusB-induced conformational change and dynamics in EF-G as the basis for its biological mechanism. We have identified two types of effect occurring in EF-G upon complexation with FusB; alterations in inter- and intra-domain conformation at the C-terminus of the protein, and a change in the conformational flexibility of domain III. The latter effect may represent a direct consequence of the former, with FusB-induced conformational change in domains IV and V acting to disrupt the interfaces between these domains and domain III, thereby releasing this intrinsically dynamic domain from restraint. Conformational change is central to the function of EF-G; comparison of structures of this protein determined in the apo form^17^,^18^, nucleotide-bound form^19^,^20^, and resident on the ribosome pre- and post-translocation^21^,^22^,^38^, show that EF-G undergoes major structural rearrangements throughout the translocation cycle, most of which involve domains I and II moving relative to domains III-V^39^. For EF-G to dissociate from the ribosome once translocation has occurred, conformational change upon GTP hydrolysis is transmitted from domains I and II to domain IV to disrupt the contacts that this latter domain makes with the 30S subunit of the ribosome^22^. Dissociation of EF-G from the ribosome is inhibited by FA, which binds to EF-G between domains II and III, and apparently restricts this transmission of conformational change^21^. FusB-induced conformational change in domains IV-V of EF-G and altered dynamics in domain III presumably predispose EF-G to release from the ribosome, and thereby act to accelerate dissociation of EF-G from the ribosome in both the absence and presence of FA.

How does FusB-induced conformational change in EF-G facilitate release of the latter from the ribosome? One possibility is that the minor structural rearrangements occurring in domains IV and V of EF-G upon complexation with FusB directly impact the interaction of the protein with the ribosome, and are alone sufficient to favor dissociation. Alternatively, FusB may drive release of EF-G from the ribosome as a consequence of allosterically triggering dynamic motion in domain III. Domain III acts as a central hub to relay conformational change between domains I-II and IV-V of the protein^21^,^39^,^40^,^41^,^42^, and therefore plays a crucial role in the transmission of structural rearrangement effecting dissociation of EF-G from the ribosome. By prompting changes in the dynamics of domain III, FusB might allow the C-terminal domains of EF-G to more readily adopt the conformation relative to domains I and II required for release, without the requirement for transmission of conformational change from domains I and II (Fig. 6). Future studies will focus on distinguishing these two possibilities.

## Material and Methods

Additional details are available in SI Materials and Methods.

### Protein expression and purification

FusB, EF-G and EF-G_C3_ were expressed and purified as previously described^14^. FusB and EF-G_C3_ were both produced with either ^15^N, ^13^C and partial ^2^H labelling, ^15^N, partial ^2^H labelling or no specific labelling while EF-G was produced with ILVA methyl ^13^C, ^1^H labelling on a uniformly ^12^C, ^2^H background, as detailed in supplementary materials and methods. Selective amino acid unlabelling was achieved by adding 1g/l of a single amino acid to ^15^N ^2^H autoinduction medium as described^43^.

### NMR Spectroscopy

NMR experiments were performed at 25 °C on either an Agilent Inova 600 MHz spectrometer with a room temperature probe, a Varian Inova 750 MHz spectrometer with a cryogenic probe or a Varian Inova 900 MHz spectrometer with a cryogenic probe (UK Biomolecular NMR facility, Birmingham). Samples for backbone assignment were produced using 0.3 mM ^15^N, ^13^C partially-deuterated EF-G_C3_ whilst samples for RDC and PRE measurements were produced using 0.3 mM ^15^N partially-deuterated FusB or EF-G_C3_. Samples of full length EF-G were prepared using 50 μM ^15^N perdeuterated EF-G with ^13^C-^1^H labelled methyl groups of alanine, leucine, valine and isoleucine. For studies of either protein in the complex the labelled protein was saturated with 1.5× the concentration of the non-isotopically enriched binding partner. All experiments were performed using TROSY modifications^44^, and deuterium decoupling where required. Data were processed in NMRPipe^45^ before assignment and measurement of CSPs using CCPN analysis^46^. Peak intensity measurements for calculation of ARTSY-based RDCs, PREs and solvent PREs were made using NMRView^47^.

#### Backbone assignment

Backbone assignments of EF-G_C3_ in the apo and complex state were determined from analysis of HNCA, HNCO, HN(CO)CA, HN(CA)CO, HNCACB and HN(CO)CACB spectra with TROSY modifications. These assignment spectra were supplemented using selectively unlabelled^43^ samples in which ^15^N EF-G_C3_ was enriched with a single non-isotopically enhanced amino acid (alanine, asparagine, lysine, valine and phenylalanine). Assignments of FusB bound to EF-G_C3_ were transferred from the apo spectra by visual inspection with the use of a series of spectra of single amino acid selectively unlabelled lysine, leucine, phenylalanine, valine and asparagine ^15^N FusB bound to EF-G_C3_^43^.

#### Chemical shift perturbation analysis

^1^H-^15^N chemical shift perturbation analysis was performed using direct calculation for residues assigned in both spectra and then using conservative chemical shift perturbation analysis for all other residues, finding the closest peak in unassigned spectrum to the assigned peaks in the apo spectrum^48^. The chemical shift change was calculated using the metric 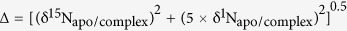
48. A cut-off for chemical shift changes of 1.0 ppm was applied for differences between EF-G_C3_ in the apo and FusB bound states, 0.6 ppm for differences between FusB in the apo and EF-G_C3_ bound states and 0.4 ppm for differences between EF-G_C3_ and EF-G. The cut-off for FusB was chosen to be consistent with previously published data^14^ while the remaining cut-offs were chosen to class any change greater than 1 standard deviation above the mean chemical shift difference as significant.

#### Amide RDC measurements

Amide RDC measurements in apo FusB and EF-G_C3_ were measured using ^15^N labelled partially deuterated protein in 5% polyacrylamide gels compressed to ⅔ original height by a Shigemi tube plunger. RDCs were quantified from the difference in position between peaks in TROSY and ^15^N semi-TROSY spectra. RDCs for FusB and EF-G_C3_ in the complex were measured using two protein samples: (i) ^15^N, partially ^2^H labelled EF-G_C3_ in complex with unlabelled FusB and (ii) ^15^N, partially ^2^H labelled FusB in complex with unlabelled EF-G_C3_. Each sample was aligned in 6 mg/ml Pf1 phage and RDCs recorded using the 2D ARTSY pulse sequence^49^. 71 and 41 ^15^N, ^1^H RDCs in the range –28.5 to +19.4 Hz were obtained for EF-G_C3_ and FusB samples respectively using only residues within secondary structure elements. Data were fitted to the crystal structures using PALES^50^ and the quality of the fit assessed through the Q factor and correlation coefficients resulting from this fit. Q_free_ values were calculated by omitting 10% RDCs (the free set) from the calculation, and then comparing the free set RDCs with values back-calculated from the refined model. The Q_free_ calculations were repeated eight times, and an average Q_free_ was calculated over all repeats.

#### PRE measurements

For PRE measurements, pET-28a: *fusB*^14^ was modified to encode FusB independently harboring amino acid substitutions R_19_C and T_26_C in the N-terminal domain, and N_150_C in the C-terminal domain (Genscript) which were labelled using MTSL as described in supplementary materials and methods. Each substitution was at a solvent exposed, non-conserved residue. ^15^N-TROSY-HSQC spectra of the mutant proteins established that these amino acid substitutions did not perturb the structure of FusB, except in the immediate vicinity of the substitution (Supplementary Fig. 7). Amide ^1^H PRE effects were made from the ratio of peak intensities from ^15^N-TROSY-HSQC spectra measured from ^15^N, partially deuterated EF-G_C3_ bound to MTSL tagged FusB mutants and ^15^N, partially deuterated EF-G_C3_ bound to untagged FusB as sample degradation prevented the use of reduced MTSL tagged samples.

#### Solvent PREs

Solvent PREs from amide proton *R*_1_ relaxation measurements were measured for ^15^N deuterated EF-G_C3_ bound to non-isotopically enriched FusB using 0.0, 0.25, 0.5, 0.75 and 1.0 mM Gd-DPTA-BMA as the probe and following the method of Madl *et. al*.^26^, as detailed in supplementary materials and methods.

### Structure calculation

#### Modelling of conformational changes

To model conformational changes upon complexation, the crystal structures were refined to better fit the RDC and solvent PRE data using Xplor-NIH^34^ and the method of Wang *et. al*.^29^. For refinement of the EF-G_C3_ domain IV internal conformation, the structure of all three domains excepting the domain IV helices and the loops joining them to the remainder of the structure was fixed while the helices were allowed to move as a rigid body. For realignment of domains IV and V of EF-G_C3_, domain IV was fixed while domain V was allowed to move as a rigid body with restraints included from RDCs from both domains. Domain III was allowed to move unrestrained as a rigid body to prevent it from sterically hindering domain realignment. To maintain a compact protein structure, solvent PRE restraints were included in all EF-G_C3_ calculations using the method of Wang *et. al*.^29^. Calculation of an ensemble of 100 structures converged well with this model with an average RMSD from the lowest energy structure for Cα atoms of 0.38 Å for the best 50 structures. For realignment of the two domains of FusB, the C-terminal domain was fixed and the N-terminal domain allowed to move as a rigid body. Calculation of an ensemble of 100 structures converged well with this model with an average RMSD from the lowest energy structure for Cα atoms of 0.08 Å for the best 50 structures.

#### Docking calculations

Docking of the structure of EF-G_C3_ with realigned domains IV and V and that of FusB with realigned domains was performed using HADDOCK^35^. Interaction surfaces were defined by ambiguous interaction restraints (AIRs) determined from those residues showing significant chemical shift perturbation on binding that were solvent exposed in the crystal structures. Orientational information for the two proteins was provided by the inclusion of RDCs from EF-G_C3_ domains IV and V and full length FusB. To compensate for differences in the degree of alignment between samples, the RDCs from EF-G_C3_ were scaled by a factor of 2.4. NOE style distance restraints were included using intermolecular PRE data, with residues showing an I_ox_/I_red_ greater than 0.9 defined as 25 Å or greater from the MTSL tag. Residues with an I_ox_/I_red_ less than 0.1 were defined as 15 Å or less from the MTSL tag. PREs with intermediate values (I_ox_/I_red_ >0.1 and I_ox_/I_red_ <0.9) were not included in the docking calculation, but were subsequently compared to values calculated from the docked model to verify the quality of the complex structure. The numbers of each distance restraint included in structure refinement and docking are shown in supplementary Table 1. Theoretical PREs from the final model were then compared to all experimental values to further validate the structural model.

All figures of protein structures were produced using the PyMOL Molecular Graphics System, Version 1.1, Schrodinger, LLC.

### Molecular dynamics simulations

All MD simulations were performed using GROMACS (version 4.6.5)^36^ using EF-G (PDB code 2XEX) as a starting point for the simulations. Domains III to V (residues 401 to 693) were taken from the structure by removing the rest of the protein, and missing residues 442–444 were rebuilt using Xplor-NIH^34^. The starting structure was solvated using the SPC water model; the thickness of the explicit water layer was at least 1.0 nm from the protein molecule. The systems were neutralized with Na^+^ and Cl^−^ ions and were equilibrated before and after insertion of ions. Production simulations were performed at constant temperature, pressure and number of particles. The all-hydrogen force field CHARMM27 was used for the simulations. Temperature and pressure were controlled using Berendsen weak coupling. All bonds with hydrogen atoms were constrained by the LINCS algorithm. Coulomb interactions were computed with the PME method. 10 Å cut-offs were used for the electrostatic and van der Waals interactions; a neighbor list for non-bonded interactions was included based on a cut-off of 10 Å. The simulation was run for 100 ns; the last 90 ns were taken for the analysis; the analysis was performed using standard procedures (*g_rms*, *g_dist, g_covar, g_anaeig)* from the GROMACS package.

### Data deposition

NMR chemical shift assignments for apo EF-G_C3_ have been deposited in the BMRB (entry number 25368), as have assignments and structural restraints for the FusB•EF-G_C3_ complex (entry number 25504). The structural model of the FusB•EF-G complex has been deposited in the PDB (PDB ID 2MZW).

## Additional Information

**How to cite this article**: Tomlinson, J. H. *et al*. A target-protection mechanism of antibiotic resistance at atomic resolution: insights into FusB-type fusidic acid resistance. *Sci. Rep*. **6**, 19524; doi: 10.1038/srep19524 (2016).

## Supplementary Material

Supplementary Information

## Figures and Tables

**Figure 1:**
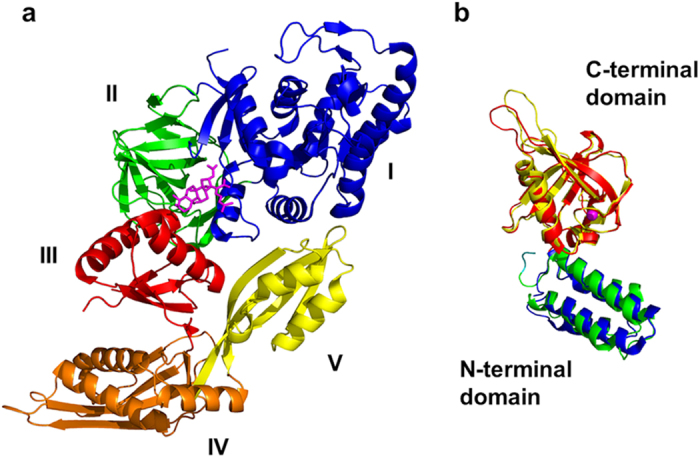
Structures of *S. aureus* EF-G and FusB-type proteins. (**a**) *S. aureus* EF-G (PDB ID 2XEX), annotated by domain, and shown with fusidic acid (magenta sticks) modelled in the binding site through alignment with the structure of ribosome-bound, FA-bound EF-G from *Thermus thermophilus* (PDB ID 2WRI). (**b**) FusB-type proteins, FusB (PDB ID 4ADN, colored blue and red) and FusC (PDB ID 2YB5, colored green and yellow) annotated by domain. The coordinated zinc ion in the C-terminal domain is shown as a magenta sphere.
