# Supplementary material for: A target-protection mechanism of antibiotic resistance at atomic resolution: insights into FusB-type fusidic acid resistance
Source: Sci Rep. 2016 Jan 19;6:19524. doi: 10.1038/srep19524 (PMC4725979; doi:10.1038/srep19524)
Supplement: Supplementary Information [file srep19524-s1.pdf]

A target-protection mechanism of antibiotic resistance at atomic resolution: insights into FusB-type fusidic acid resistance

Jennifer H. Tomlinson, Gary S. Thompson, Arnout P Kalverda, Anastasia Zhuravleva and Alex J. O'Neill*

Supplementary Information

SUPPLEMENTARY MATERIALS AND METHODS

Protein expression and purification

FusB, EF-G and EF-G_{C3} were expressed and purified as previously described.[1] ^{15}N, partially deuterated and non-isotopically enhanced proteins were produced by the auto-induction method[2] while ^{13}C, ^{15}N, partially deuterated and ILVA-^{13}C, ^{15}N perdeuterated proteins were produced by IPTG induced over expression at 18 °C overnight. ^{15}N, partially deuterated proteins were produced by growth in 100 % D_2O minimal auto-induction medium[2] containing 2.5 g/l ^{15}N ammonium chloride at 25 ºC with aeration for 72 hours. ^{15}N, ^{13}C partially deuterated proteins were produced by growth in 100 % D_2O M9 minimal medium containing 2.5 g/l ^{13}C glucose and 1 g/l ^{15}N ammonium chloride at 37 ºC with aeration until the OD_{600nm} reached 0.6 followed by induction with 1 mM IPTG and incubation at 18 ºC overnight with aeration. Unlabelled proteins were expressed using unlabelled auto-induction medium[2] at 25 ºC for 48 hours with aeration. ^{15}N, ^{12}C, ^{2}H isoleucine Cδ1, leucine Cδ, valine Cγ and alanine Cβ methyl ^{13}C, ^{1}H labelled EF-G was produced by growth in 100 % D_2O M9 minimal medium as above but with ^{12}C-^{2}H glucose at 37 ºC with aeration until the OD_{600nm} reached 0.5. 2.5 g/l $^{2}H_4$ succinic acid, 0.12 g/l methyl-^{13}C, 3,4,4,4,-$^{2}H_4$ α-

ketoisovaleric acid, sodium salt, 0.06 g/l methyl-^{13}C, 3,3-$^{2}H_2$ α-ketobutyric acid, sodium salt and 0.8 g/l 3-^{13}C, 2-^{2}H L-alanine were added and growth continued for 40 minutes. Protein expression was then induced by addition of 1 mM IPTG and incubation at 18 ºC overnight with aeration. Cultures were harvested by centrifugation at 4200 ×g, 4 ºC for 30 minutes.

All proteins were purified by nickel affinity chromatography followed by overnight dialysis and gel filtration at 4 ºC as previously described.[1] Protein samples were prepared in 20 mM trisHCl, 300 mM NaCl, 1 mM DTT, 10 %D_2O, pH 8.0 except for PRE samples for which the DTT was omitted.

NMR Spectroscopy

Backbone assignments of EF-G_{C3} were determined from analysis of TROSY-HNCA, HNCO, HN(CO)CA, HN(CA)CO, HNCACB and HN(CO)CACB spectra. For assignment of EF-G_{C3} bound to FusB, spectra were limited to the HNCO, HNCA and HN(CO)CA coupled to comparison with the apo state assignments due to technical limitations caused by the molecular size and the limited sample concentration available, higher concentrations promoting aggregation. These assignment spectra were supplemented using selectively unlabelled[3] samples in which ^{15}N EF-G_{C3} was enriched with a single non-isotopically enhanced amino acid (alanine, asparagine, lysine, valine and phenylalanine). Assignments of FusB bound to EF-G_{C3} were transferred from the apo spectra by visual inspection with the use of spectra of selectively unlabelled lysine, leucine, phenylalanine, valine and asparagine ^{15}N FusB bound to EF-G_{C3}.

^{1}H-^{15}N chemical shift perturbation analysis for EF-G$_{C3}$ was performed using only residues assigned in both the apo and FusB bound spectra. Conservative chemical shift perturbation analysis to compare EF-G$_{C3}$ and EF-G was performed by finding the closest peak in the EF-G spectrum to the assigned peaks in the EF-G$_{C3}$ spectrum.[4] For FusB binding to EF-G$_{C3}$, analysis was performed as for EF-G$_{C3}$ for residues assigned in both spectra and then using the conservative closest peak method for the remainder of the apo FusB assignments. The chemical shift change was calculated using the metric $\Delta = \left[\left(\delta^{15}\mathrm{N}_{\mathrm{apo/complex}}\right)^2 + \left(5 \times \delta^{1}\mathrm{H}_{\mathrm{apo/complex}}\right)^2\right]^{0.5}$ [4]. A cut-off for chemical shift changes of 1.0 ppm was applied for differences between EF-G$_{C3}$ in the apo and FusB bound states, 0.6 ppm for differences between FusB in the apo and EF-G$_{C3}$ bound states and 0.4 ppm for differences between EF-G$_{C3}$ and EF-G.

For PRE measurements, pET-28a:*fusB* was modified to encode FusB independently harbouring amino acid substitutions R$_{19}$C, T$_{26}$C, and N$_{150}$C (Genscript). Purified mutant proteins were mixed with 3 molar equivalents of MTSL in 20 mM TrisHCl, 300 mM NaCl (pH 8.0) for 2 hours before buffer exchange and concentration in vivaspin-6 5000 kDa concentrators at 4500 ×*g*. Amide ^{1}H PRE measurements were made from the ratio of peak intensities from ^{15}N-TROSY-HSQC spectra measured from ^{15}N deuterated EF-G$_{C3}$ bound to MTSL tagged FusB mutants and ^{15}N deuterated EF-G$_{C3}$ bound to untagged FusB as sample degradation prevented the use of reduced MTSL tagged samples.

Solvent PREs from amide proton R_1 relaxation measurements were measured for ^{15}N deuterated EF-G$_{C3}$ bound to non-isotopically enriched FusB using Gd-DPTA-BMA as the probe and following the method of Madl *et. al.*.[5] Amide proton R_1 relaxation

measurements were performed using the saturation recovery method in the presence of 0.0, 0.25, 0.5, 0.75 and 1 mM Gd-DPTA-BMA (a kind gift from Tobias Madl). The slope of a linear fit of these rates plotted against Gd-DPTA-BMA concentration was taken as the PRE value.

For determination of ^{1}H R_1 values to allow calculation of solvent PREs, saturation recovery T_1 relaxation series of TROSY-HSQCs with delays of 0.2, 0.6, 1.0, 1.5, 2.0, 4.0 and 6.0 s were run in an interleaved experiment with delay times randomised to avoid systemic errors and with repeated 2.0 and 4.0 s delay times. These data were fitted to a decreasing exponential curve with the formula $\mathrm{I} = \mathrm{I}_0\left(1 - \mathrm{e}^{(-\mathrm{t}/R_1)}\right)$ where I = intensity, I_0 = intensity at zero relaxation delay and t = delay time.

Computational approaches

Where PALES fitting of RDC data indicated realignment of elements of the protein structure in the bound state, the crystal structure was refined to better fit the RDC data using Xplor-NIH.[6] To maintain a compact protein structure, solvent PRE restraints were included using the method of Wang *et. al.*.[7] For realignment of the domain IV helices, the structure of all three domains excepting the helices in question and the loops joining them to the remainder of the structure was fixed while the helices were allowed to move as a rigid body.

For realignment of domains IV and V of EF-G_{C3}, domain IV was fixed while domain V was allowed to move as a rigid body with restraints included from RDCs from both domains. Domain III was allowed to move unrestrained as a rigid body to prevent it from sterically hindering domain realignment as the broadening of domain III peaks

means no data exists to determine the orientation of domain III when bound to FusB. Calculation of an ensemble of 100 structures converged well with this model with an average RMSD from the lowest energy structure for Cα atoms of 0.38 Å for the best 50 structures.

For realignment of the two domains of FusB, the C terminal domain was fixed and the N terminal domain allowed to move as a rigid body to refine the structure to the RDCs. This calculation did not include solvent PRE effects. Calculation of an ensemble of 100 structures converged well with this model with an average RMSD from the lowest energy structure for Cα atoms of 0.08 Å for the best 50 structures.

Docking of the structure of EF-G_{C3} with realigned domains IV and V and that of FusB with realigned domains was performed using HADDOCK.[8] Interaction surfaces were defined by ambiguous interaction restraints (AIRs) determined from those residues showing significant chemical shift perturbation on binding that were solvent exposed in the crystal structures. Orientational information for the two proteins was provided by the inclusion of RDCs from EF-G_{C3} domains IV and V and full length FusB. NOE style distance restraints were included using intermolecular PRE data, with residues showing an I_{ox}/I_{red} greater than 0.9 defined as 25 Å or greater from the MTSL tag. Residues with an I_{ox}/I_{red} less than 0.1 were defined as 15 Å or less from the MTSL tag. The numbers of each distance restraint included in structure refinement and docking are shown in supplementary table 1 and a summary of the refinement statistics are shown in supplementary table 2.

Supplementary table 1. The restraints used in structure calculations.

NMR distance restraints	EF-G_{C3}	FusB
Ambiguous distance restraints from CSPs	16	20
RDCs	71	41
PREs	137	-
Solvent PREs	102	-

Supplementary table 2. Refinement statistics

Structure statistics	
Violations (mean and s.d.)	
Distance constraints (Å)	2.33 ± 1.13
Max. distance constraint violation (Å)	4.94
Deviations from idealized geometry	
Bond lengths (Å)	0.0042 ± 0.0004
Bond angles (°)	0.61 ± 0.03
Impropers (°)	0.56 ± 0.04
Average pairwise RMSD over 200 structures (Å)	0.70 ± 0.17

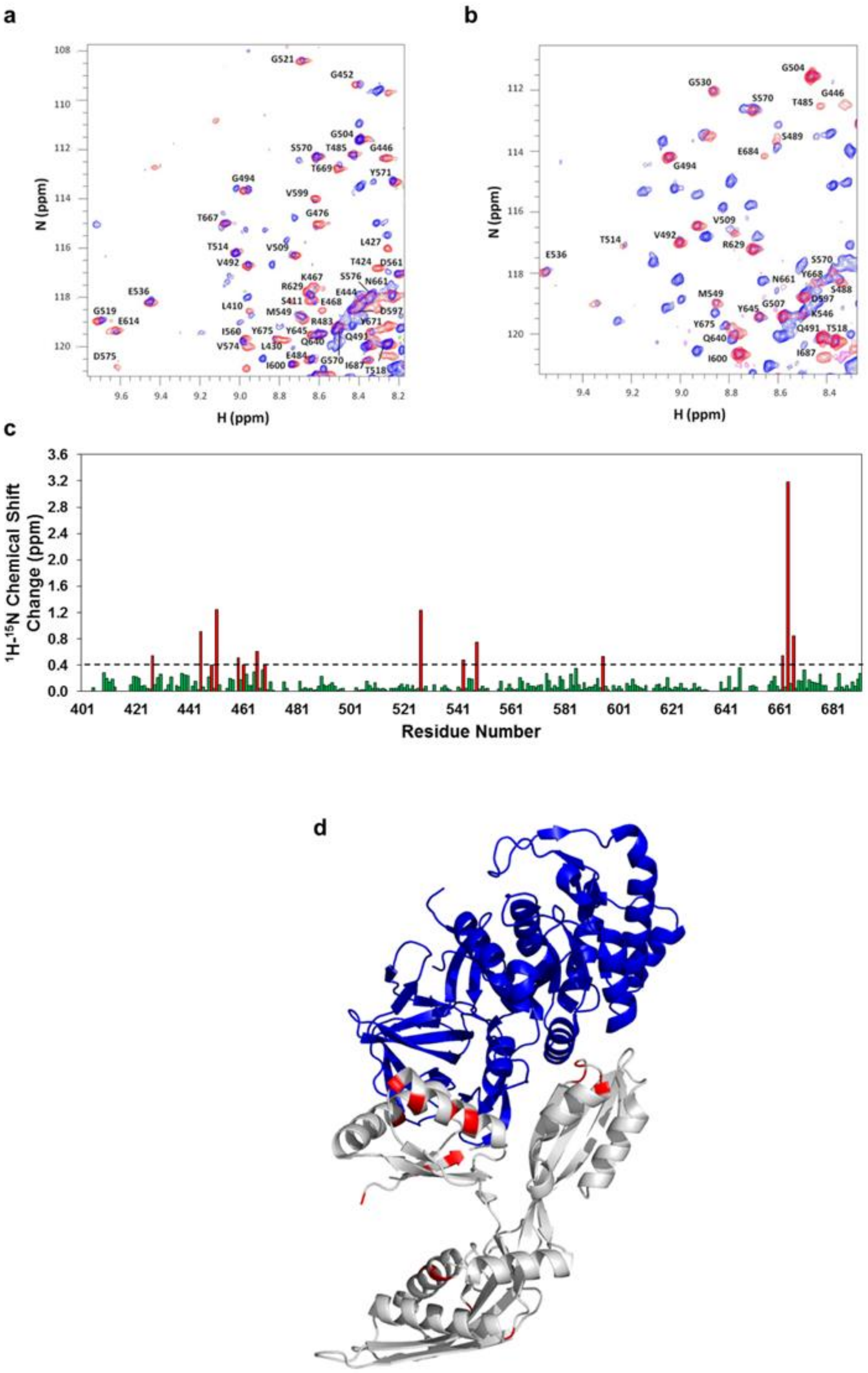

Supplementary fig. 1. Comparison of EF-G$_{C3}$ and EF-G spectra to determine any significant chemical shift changes and show dynamics in domain III of EF-G$_{C3}$ is not an artefact of truncation. **(a and b)** Comparison of amide HSQC spectra of EF-G$_{C3}$ (red) and full length EF-G (blue) in the apo state **(a)** and bound to FusB **(b)**. (**c)** Minimal chemical shift differences calculated for residues in domains III-V between EF-G$_{C3}$ and EF-G in the apo state. Significant chemical shift changes, above 0.4 ppm, are shown in red. **(d)** Significant chemical shift differences between EF-G and EF-G$_{C3}$ in the apo state

highlighted on the crystal structure in red. Domains I and II are coloured blue. Shift differences are mainly confined to the interface between the truncated protein and domains I and II. Those seen in domain IV are most likely the result of loss of sensitivity preventing detection of the peak in the EF-G spectrum rather than genuine chemical shift differences.

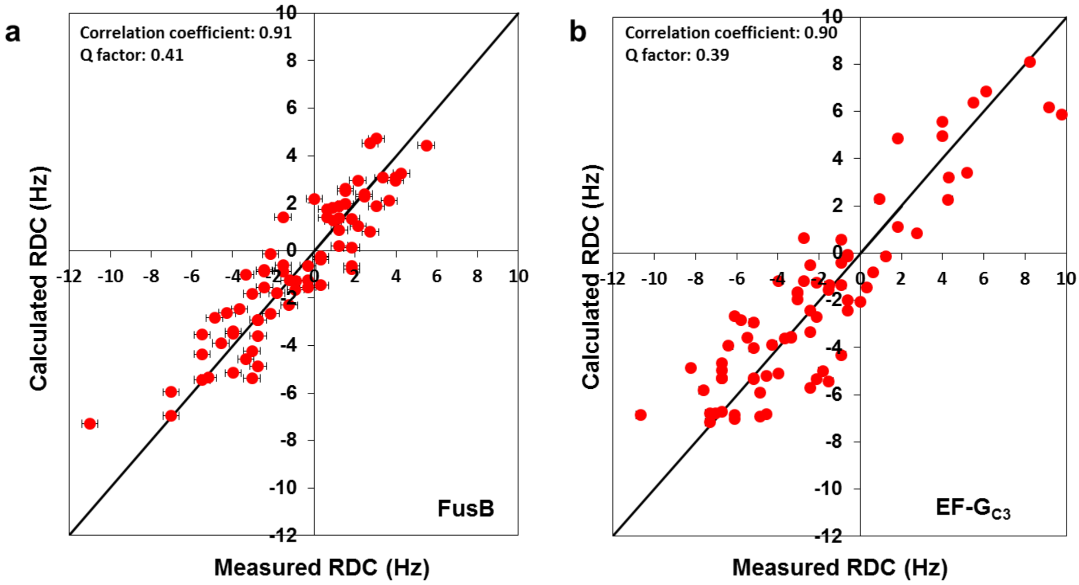

Supplementary fig. 2. Comparison of measured amide RDCs to those calculated from the crystal structures for **(a)** apo FusB and **(b)** apo EF-G$_{C3}$. Only RDCs from within secondary structure regions were included in the fits. Where error bars for the measured data are not visible, errors lie within the confines of the data points.

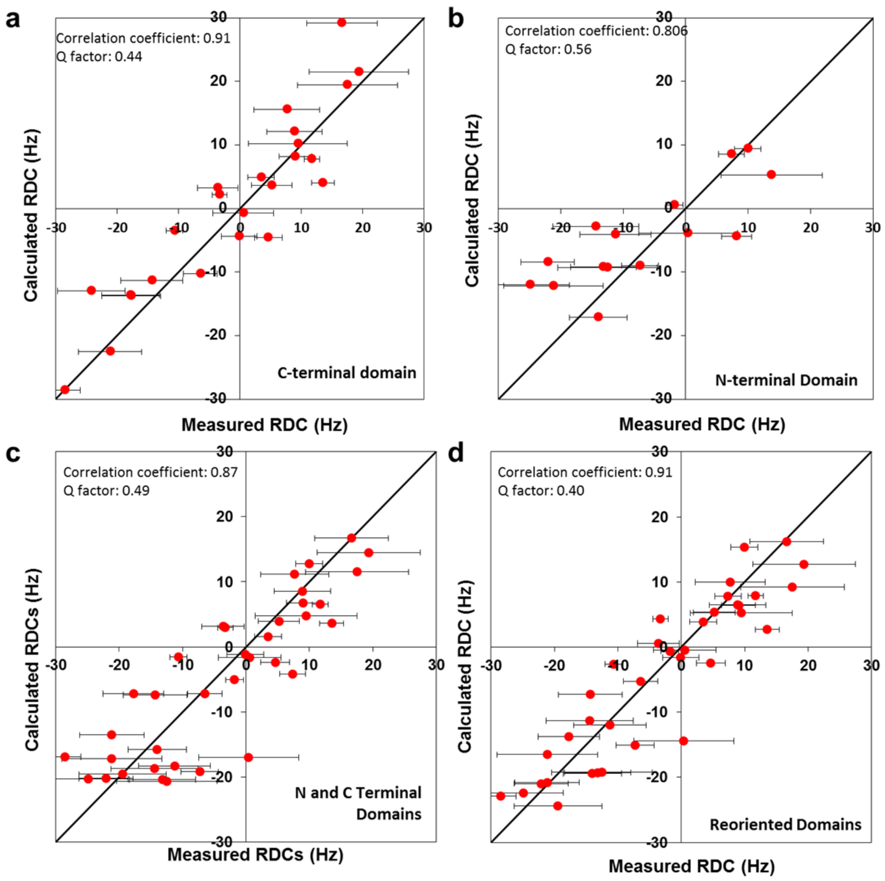

Supplementary fig. 3. Comparison of amide RDCs measured in FusB bound to EF-G_{C3} with those calculated from the crystal structure for **(a)** the C-terminal domain, **(b)** the N-terminal domain, **(c)** both domains fitted together to the crystal structure and **(d)** both domains after domain reorientation. The RDCs calculated from the structure after domain reorientation show a better fit to the observed data than those calculated from the crystal structure.

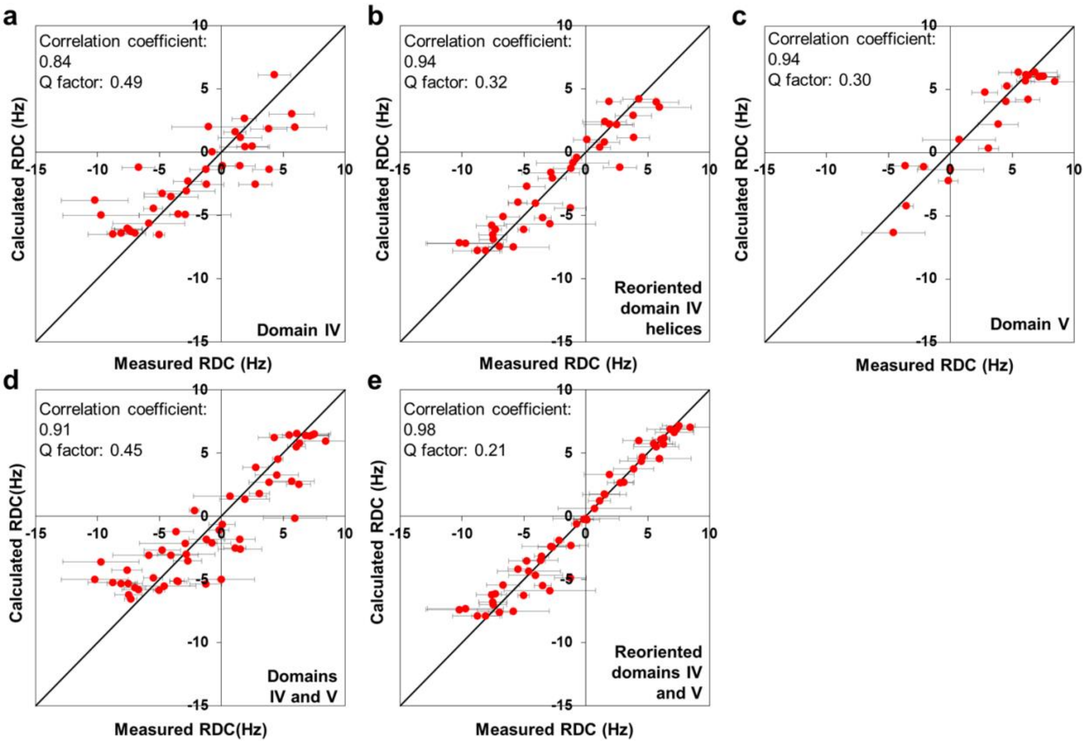

Supplementary fig. 4. Comparison of amide RDCs measured in FusB bound EF-G_{C3} with those calculated from the crystal structure for **(a)** domain IV, **(b)** domain IV after refining the positions of the helices, **(c)** domain V, **(d)** both domains IV and V fitted together and **(e)** both domains IV and V after modelling the change in relative orientation of the two domains. RDCs from residues within loop regions were omitted in each case. The RDCs calculated from the structure after domain reorientation show a better fit to the observed data than those calculated from the crystal structure.

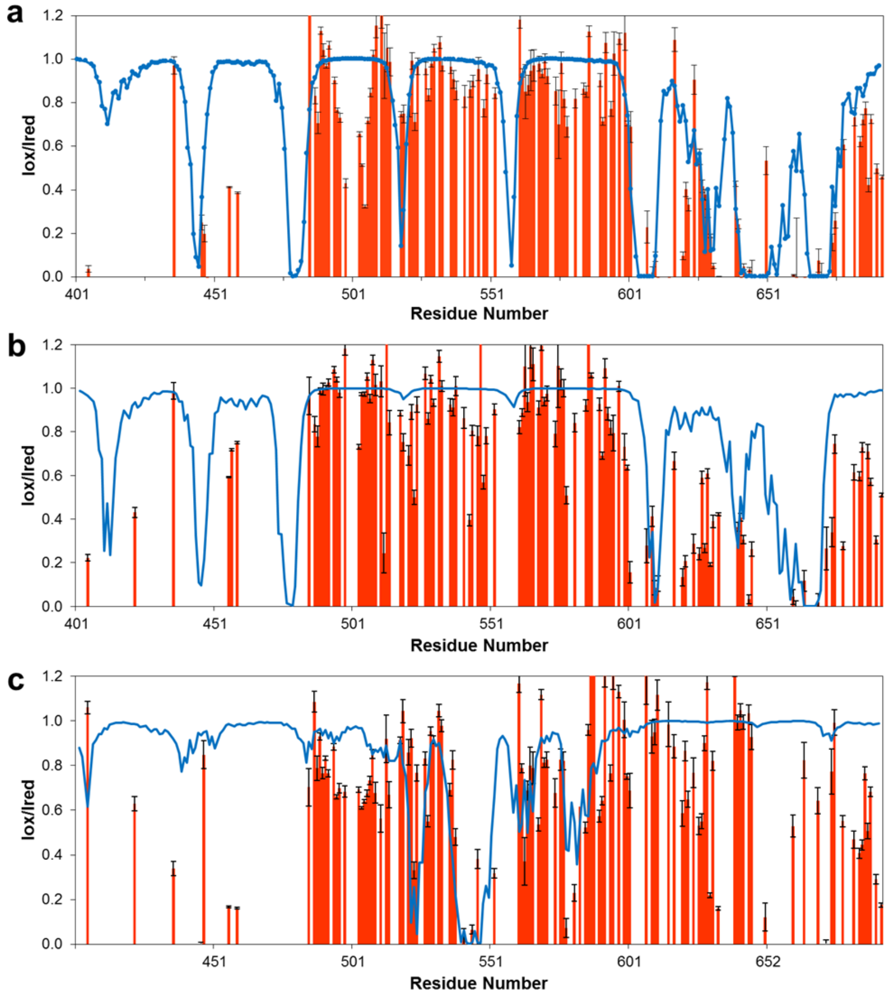

Supplementary fig. 5. Comparison of PRE effects in EF-G_{C3} from bound MTSL tagged FusB mutants to those calculated from the structural model of the complex. **(a)** PREs to FusB-R_{19}C-MTSL. **(a)** PREs to FusB-T_{26}C-MTSl. **(b)** PREs to FusB-N_{150}C-MTSL.

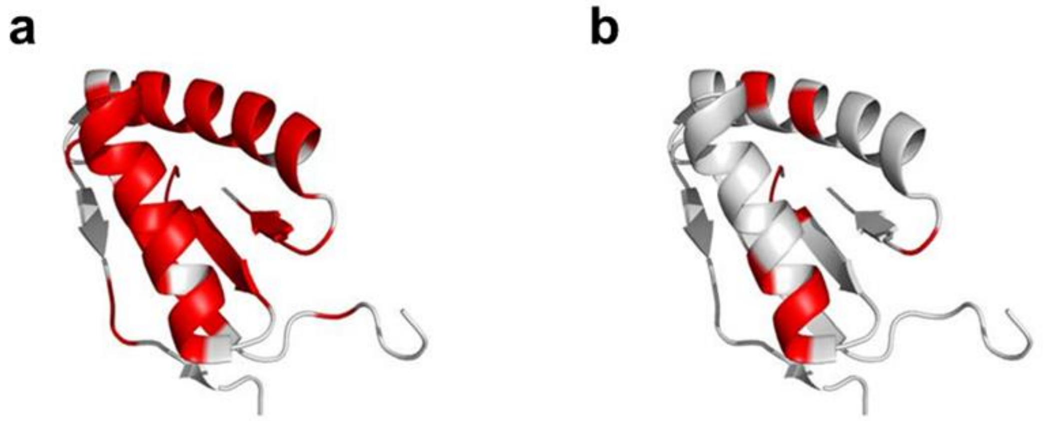

Supplementary fig. 6. The extent of assignments in domain III (residues 401-480) of EF-GC3. Assigned residues are shown in red for (**a**) apo EF-G_{C3} and (**b**) EF-G_{C3} bound to FusB on domain III of the crystal structure of apo EF-G (PDBID 2XEX).

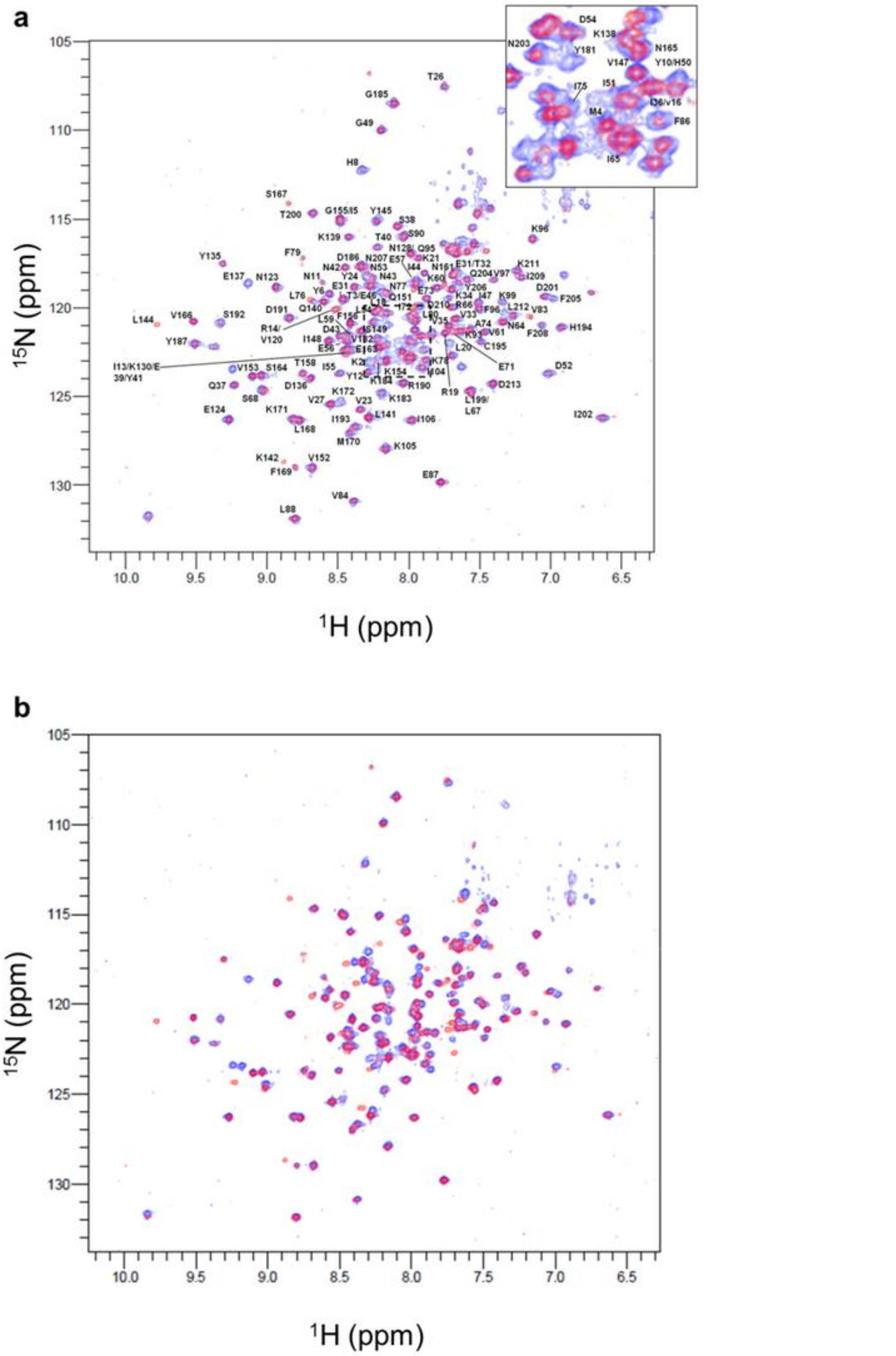

Supplementary fig. 7. Comparison of mutant and wild-type FusB spectra showing no perturbation of protein structure. Comparison of 15N TROSY-HSQC spectra of **(a)** FusB-K70C and **(b)** FusB-R19C (blue) to wild type FusB (red). The mutations did not cause chemical shift perturbation except in the immediate vicinity of the mutation, indicating the protein structure was not disrupted. Resonance assignments are shown in **(a)**, the inset panel of which shows the area inside the dashed markings magnified for clarity.

Supplementary Information References

1 Cox, G. *et al.* Ribosome clearance by FusB-type proteins mediates resistance to the antibiotic fusidic acid. *Proc. Natl. Acad. Sci. USA* **109**, 2102-2107 (2012).

2 Studier, F. W. Protein production by auto-induction in high density shaking cultures. *Protein Expr. Purifi.* **41**, 207-234 (2005).

3 Atreya, H. S. & Chary, K. V. R. Selective 'unlabeling' of amino acids in fractionally ^{13}C labeled proteins: an approach for stereospecific NMR assignments of CH_3 groups in Val and Leu residues. *J. Biomol. NMR* **19**, 267-272 (2001).

4 Williamson, R. A., Carr, M. D., Frenkiel, T. A., Feeney, J. & Freedman, R. B. Mapping the binding site for matrix metalloproteinase on the N-terminal domain of the tissue inhibitor of metalloproteinase-2 by NMR chemical shift perturbation. *Biochemistry* **36**, 13882-13889 (1997).

5 Madl, T., Güttler, T., Görlich, D. & Sattler, M. Structural analysis of large protein complexes using solven paramagnetic relaxation enhancements. *Angew. Chem. Int. Ed* **50**, 3993-3997 (2011).

6 Schwieters, C. D., Kuszewski, J. J. & Clore, G. M. Using Xplor-NIH for NMR molecular structure determination. *Prog. Nucl. Magn. Reson. Spectrosc.* **48**, 47-62 (2006).

7 Wang, Y., Schwieters, C. D. & Tjandra, N. Parameterization of solvent-protein interaction and its use on NMR protein structure determination. *J. Magn. Reson.* **221**, 76-84 (2012).

8 de Vries, S. J. *et al*. HADDOCK versus HADDOCK: new features and performance of HADDOCK2.0 on the CAPRI targets. *Proteins* **69**, 726-733 (2007).
